# Evaluation of Concussion Incidence and Modulating Factors in the 2013-2017 Australian Football League

**DOI:** 10.7759/cureus.3465

**Published:** 2018-10-18

**Authors:** Ryan Adams, George K Lau, Jennifer B Dai, Adam Y Li, Kevin P Cheung, Syed Haider, Alex Gometz, Alexander F Post, Tanvir F Choudhri

**Affiliations:** 1 Neurosurgery, The Icahn School of Medicine at Mount Sinai, New York, USA; 2 Miscellaneous, Touro College of Medicine, Bronx, USA; 3 Neurosurgery, Columbia University, New York, USA; 4 Physical Medicine and Rehabilitation, Concussion Management of New York, New York, USA

**Keywords:** sports concussion, concussion incidence, concussion rate, australian football league, style of play, concussion severity, contact sports, professional league injury

## Abstract

Introduction

The increasing awareness and popularization of concussions in the research realm over the last few years have begun to shed more light on the detrimental effects associated with repetitive head trauma. While the majority of the current literature focuses on the National Football League (NFL) and National Hockey League (NHL), several other high-impact sports have been implementing concussion management protocols to protect their players. The Australian Football League (AFL) is a prime example of a major contact sport that has undertaken recent changes to its concussion assessment and management modalities. We recognize the benefit of reporting potential changes in concussion rates over the 2013-2017 AFL seasons. We were interested in some of the factors not yet evaluated before, which may contribute to the overall concussion incidence such as “style-of-play” factors” (home/away, win/loss, points scored, time of season). We hope the results of this analysis shed light on the mechanisms by which concussion rates can be mitigated across major contact sports.

Methods

Data were obtained from the weekly injury list published by the AFL, a publicly available website. Details of players listed with concussions were collected from regular season games (890 games total) of 18 AFL teams from 2013 to 2017. Weekly injury lists were retrospectively compared using THE AGE, a publicly available newspaper published and owned by Fairfax Media. Data were analyzed utilizing GraphPad Prism 6 (GraphPad Software Inc., CA, US). In addition to descriptive statistics, Fisher's exact tests, Welch’s two-tailed t-tests, and correlation tests were used. Alpha level < 0.05 was considered significant for all tests.

Results

The dataset comprised 236 total concussions between the 2013 and 2017 AFL seasons. We noted a significant increase in the concussion rate (p = 0.0010) between the 2013 and 2017 seasons. This result was complemented by a significant increase in average games missed between the 2014 and 2015 seasons (p = 0.0002); however, this trend was not significant when evaluating the 2013 and 2017 seasons directly (p = 0.0951). Further analysis into the "style of play" factors on concussion incidence, such as average points scored, win/loss, home/away, and time of season, produced non-significant results.

Conclusion

Our study identified a significant increasing trend in concussion rate and average games missed that correlate to the data analysis in other high-impact sports such as the NFL and NHL. However, further research is necessary to determine if these findings indicate the improvement in concussion management and player safety measures beginning to develop in high-impact sports. We also noted that certain “style of play” factors (points scored, win/loss, home/away, and time of season) have no significant implication on concussion rate during the 2013-2017 AFL seasons. While we consider our data source to be reliable in the reporting of concussions from the AFL, the ideal data set would comprise a medical diagnosis from the team of doctors. It may be possible that our data set is underreporting the total amount of concussions between the 2013 and 2017 AFL seasons. Return-to-play times were not ascertained directly from the team doctor for the clearance date. It may be possible that this data collection modality led to missed cases of head injury or return to play times, which could impact the reliability of our dataset.

## Introduction

The increasing awareness and popularization of concussions in the research realm over the last few years have begun to shed more light on the detrimental effects associated with repetitive head trauma. Neuropsychiatric changes, cognitive impairment, motor dysfunction, and increased risk for neurodegenerative disease have all been linked to repetitive head trauma and sports-related concussions. [1-5]. The severity of these detrimental effects has given rise to a body of literature evaluating ways to mitigate their occurrence. More research has been initiated to evaluate head-impact biomechanics, rules changes to limit injury exposure, equipment viability, and the nutritional supplementation of players’ diets [6].

While the majority of the current body of literature focuses on the National Football League (NFL) and National Hockey League (NHL), several other high-impact sports have been implementing concussion management protocols in order to further protect their players The Australian Football League (AFL) is a prime example of a major contact sport that has undertaken recent changes to its concussion assessment and management modalities. Injuries are common and 38-46 new injuries per club have been recently reported per year. These injuries render up to 18% of the playing list at any club unavailable at any given time during a season. The variety of injuries tend to range from muscle strains to compound fractures and include concussions [7]. A previous study evaluated the injury rate of the AFL over a 21-year period from 1992 to 2012 and demonstrated 4492 players listed over the 21-year period who suffered 13,606 new injuries/illnesses and 1965 recurrent injuries/illnesses, resulting in 51,919 missed matches [8].

While several prior studies have been published investigating injury rates in the AFL, there is a striking lack of information dedicated purely to concussion incidence. As concussion recognition and management protocols have improved in the past few years, we recognize the benefit of reporting on potential change in concussion rate over the 2013-2017 AFL seasons. We were interested in some of the factors not yet evaluated before, which may contribute to the overall concussion incidence such as “style-of-play factors” (home/away, win/loss, points scored, time of season). We hope the results of this analysis shed light on the mechanisms by which concussion rates can be mitigated across major contact sports.

## Materials and methods

Data were obtained from the weekly injury list published by the Australian Foot League, a publicly available website. Details of players listed with concussions were collected from regular season games (890 games total) of 18 Australian Football League (AFL) team from 2013 to 2017. The weekly-injury-list was retrospectively compared using THE AGE, a publicly available newspaper published and owned by Fairfax Media.

Data collection

This retrospective study examined the 2013-2017 AFL regular seasons (five total seasons from Round 1 to Round 23). Official concussion injury reports were collected from the weekly injury report released by the Australian Football League. A retrospective comparison of the published weekly-injury-list was made using THE AGE, a publicly available newspaper published and owned by Fairfax Media.

Definition

Games Missed: The total number of regular season games a player misses due to a concussion injury, excluding the game in which they were concussed.

Style of Play Factors: Points Scored, Win/Loss, Home/Away, Time of Season

Australian Football League Season: Composed of 23 rounds, which occur on a weekly basis. We divided the season into two halves, Rounds 1-11 and 12-23.

Concussion Rate: (total number of concussions) / (total number of games per season)

Concussion rate across major contact sports

National Football League (NFL): The concussion rate acquired through a previously "in-review" work produced by our study team. PBS Frontline utilized as the source data [Original Article: Haider S, Sobotka S, Choudhri T. Role of Environmental Risk Factors in Concussion Incidence in the National Football League from 2012 to 2015: 2017].

National Hockey League (NHL): Concussion rate acquired through a previously published work produced by our study team. FOX Sports Injury Tracker utilized for source data. We acknowledge a limitation in this dataset due to a lack of publically available data pertaining to concussion injuries and consider it to underreport total concussions that occurred between the 2013 and 2017 NHL seasons [9].

Australian Football League (AFL): Concussion rate acquired through the current body of work utilizing source data from the league.

Statistical analysis

Data were analyzed utilizing GraphPad Prism 6 (GraphPad Software Inc., CA, US). In addition to descriptive statistics, Fisher's exact tests, Welch’s two-tailed t-tests, and correlation tests were used. Alpha level < 0.05 was considered significant for all tests.

## Results

AFL teams that participated in the 2013-2017 seasons and stadium locations were documented (Table 1). One important note is that several teams are based out of the same stadium. Our analysis considers this factor and we based our home/away analysis on the AFL matchday report, which clarifies which team is deemed the “home team” for that specific matchup. Due to the close proximity of each city, our research team did not consider “travel distance” or “change in altitude” between matches as analyzed in previous works.

**Table 1:** AFL Teams Competing in the 2013-2017 Seasons Australian football teams participating in the 2013-2017 seasons. Stadiums presented are representative of where the team played for the 2013-2017 season. Team abbreviation also provided.

Team Name	Abbreviation	Location	Arena
Adelaide Football Club	ADE	Adelaide, South Australia	Adelaide Oval
Brisbane Lions	BRI	Brisbane, Queensland	The Gabba
Carlton Football Club	CAR	Melbourne, Victoria	Etihad Stadium
Collingwood Football Club	COL	Melbourne, Victoria	MCG
Essendon Football Club	ESS	Melbourne, Victoria	Etihad Stadium
Fremantle Football Club	FRE	Perth, Western Australia	Domain Stadium
Geelong Football Club	GEE	Geelong, Victoria	Simonds Stadium
Gold Coast Football Club	GC	Gold Coast, Queensland	Metricon Stadium
Greater Western Sydney Giants	GWS	Western Sydney, New South Wales	Spotless Stadium StarTrack Oval
Hawthorn Football Club	HAW	Melbourne, Victoria	MCG
Melbourne Football Club	MEL	Melbourne Cricket Ground	MCG
North Melbourne Football Club	NM	Melbourne, Victoria	Etihad Stadium
Port Adelaide Football Club	PA	Adelaide, South Australia	Adelaide Oval
Richmond Football Club	RICH	Melbourne, Victoria	MCG
St Kilda Football Club	StK	Melbourne, Victoria	Etihad Stadium
Sydney Swans	SYD	Sydney, New South Wales	SCG
West Coast Eagles	WCE	Perth, Western Australia	Domain Stadium
Western Bulldogs	WB	Melbourne, Victoria	Etihad Stadium

Demographics were broken down by a season-to-season basis with 236 concussions documented in 890 total games (Table 2). We noted a significant trend pertaining to an increase in reported concussions from the 2013 through 2017 seasons (p = 0.0010). After analyzing each season, we observed that a concussion occurred in 19% of all games played. However, the vast majority of concussions were documented in the 2016 and 2017 seasons (74 and 65 concussions, respectively). While more concussions occurred from the home team perspective compared with the away team, the trend was non-significant (p = 0.6367).

**Table 2:** Demographic Data of Included Team Games Demographic analysis of concussion incidence on a season-to-season basis and home vs. away perspective. Values were produced from our finalized dataset.

Demographics Data of Included Team Games (N = 890)		
Variable	n	%
2013 season concussions	24	10
2014 season concussions	35	15
2015 season concussions	38	16
2016 season concussions	74	31
2017 season concussions	65	28
Total Number of Concussions	236	100
Home Team Concussions	129	55
Away Team Concussions	107	45
Total Games With Concussion	190	19
Total Games Without Concussion	800	81
2013 Concussion Rate	0.13	
2014 Concussion Rate	0.17	
2015 Concussion Rate	0.19	
2016 Concussion Rate	0.36	
2017 Concussion Rate	0.32	

**Figure 1:**
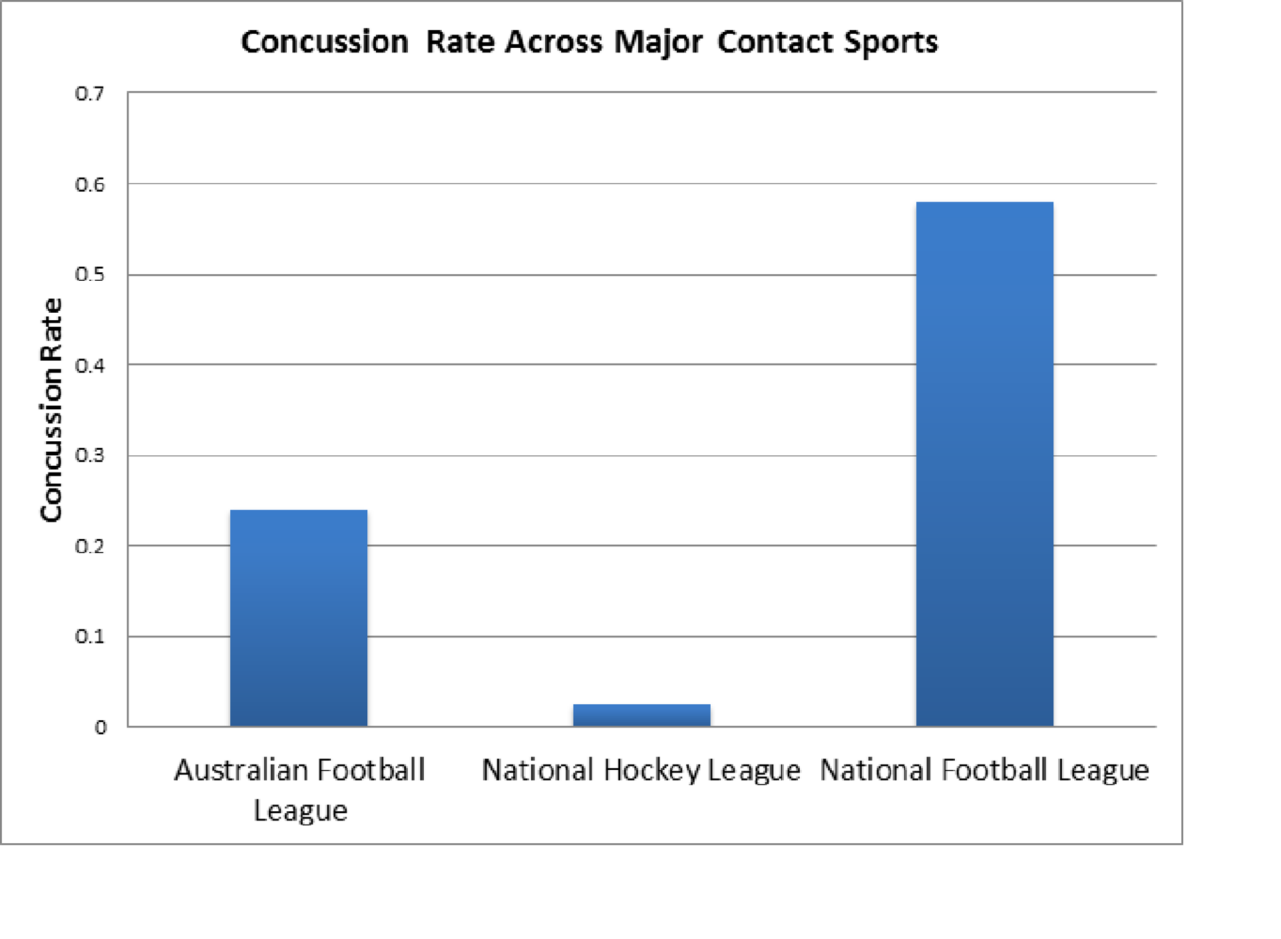
Concussion Rate Across Major Contact Sports Calculated concussion rate per game comparison between the National Hockey League (NFL), the National Football League (NFL), and Australian Football League (AFL). The NHL and NFL concussion rate was determined by our research group in previously published works.

Evaluating the concussion rate across major contact sports was a cornerstone of our research groups analysis (Figure 1). Prior publications composed by our research group discerned the average concussion rate in both the NHL and NFL. We noted an average concussion rate of 0.025 in the NHL and 0.58 in the NFL. Analysis indicated a concussion rate of 0.24 in the AFL. Due to the variations in rules, concussion management, and play style across these sports, we provided this graph of our groups' findings for observational purposes in lieu of testing for statistical significance.

**Figure 2:**
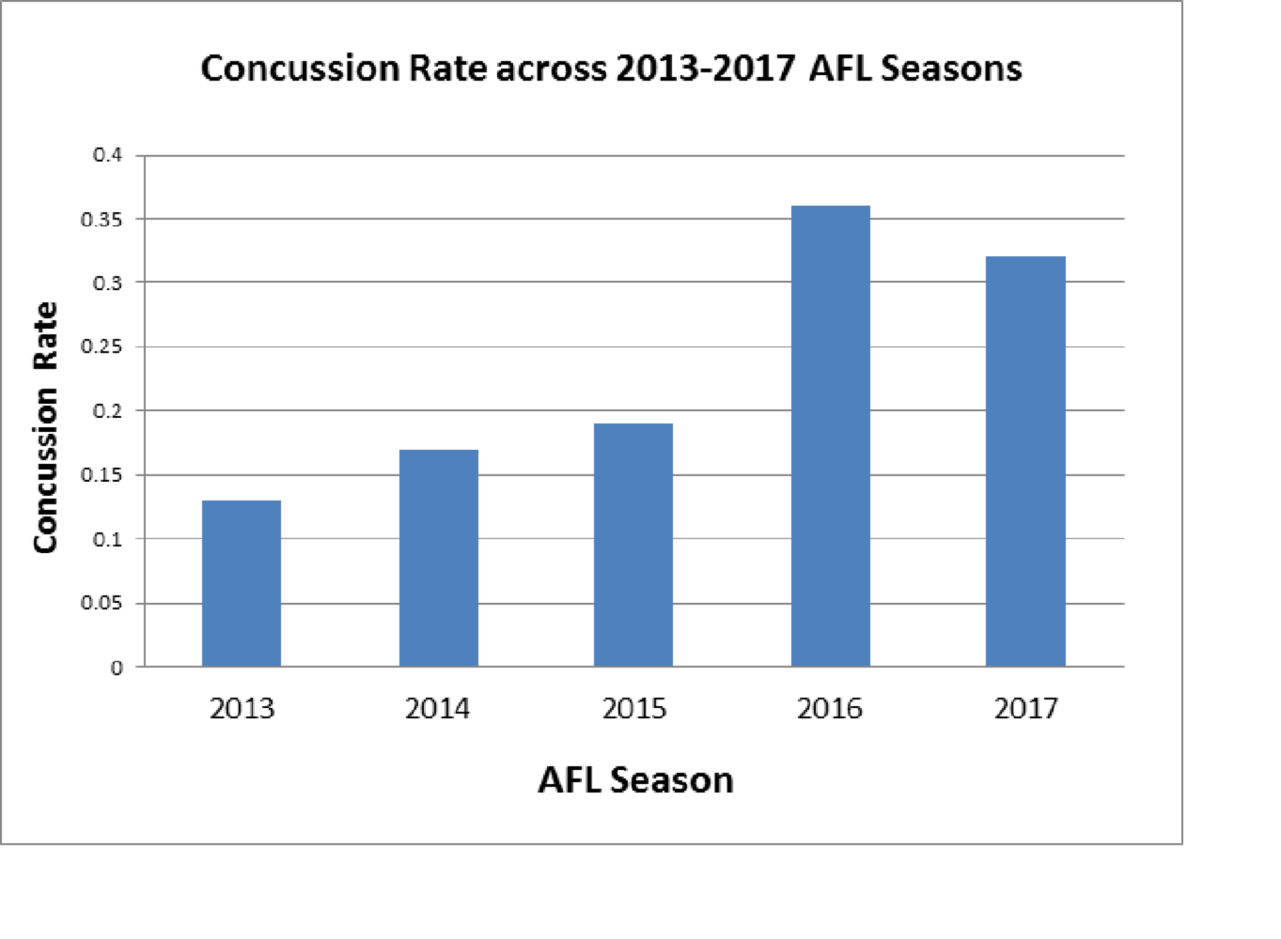
Concussion Rate Across 2013-2017 AFL Seasons Concussion rate calculated individually across the 2013-2017 Australian Football League (AFL) seasons

The concussion rate for each season was calculated and demonstrated an increasing concussion rate between 2013 and 2017 (Figure 2). There was a significant increase in concussion rate when comparing the 2013 to the 2017 AFL seasons (p = 0.0010). Overall, we noted an increasing trend towards higher concussion rates per season from the 2013 to the 2017 seasons. 

**Figure 3:**
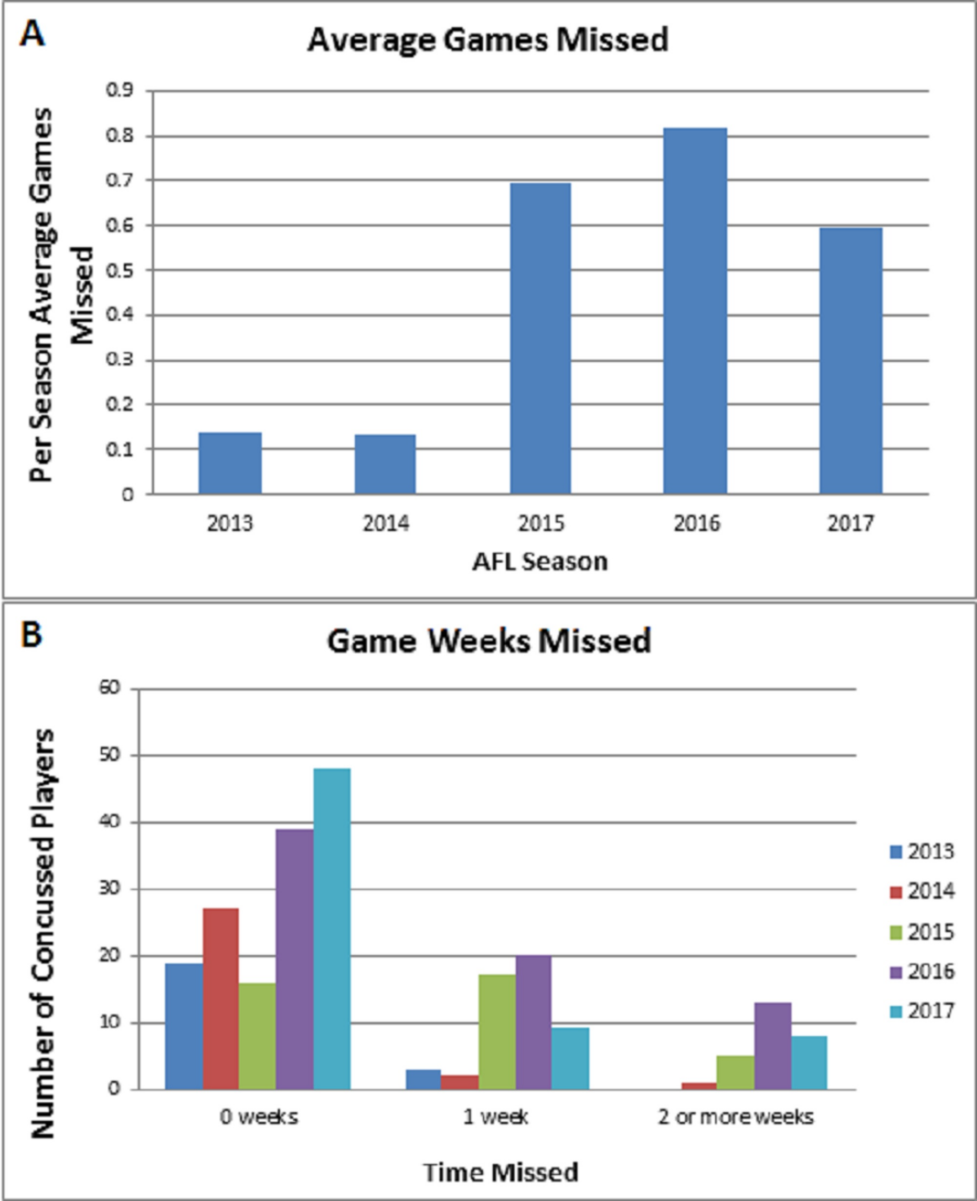
Games Missed Analysis (A) Average games missed analysis from the 2013-2017 Australian Football League (AFL) seasons, (B) Bar graph demonstrating number of player weeks missed due to concussion for the 2013-2017 AFL seasons. Note that our dataset contained no concussions resulting in >2 weeks missed for the 2013 season.

The average games missed analysis demonstrated a significant difference between the 2014 and 2015 AFL seasons (p = 0.0002). However, this significant trend was not noted when comparing the 2013 and 2017 seasons (p = 0.0951) (Figure 3A). The majority of concussed players also missed no subsequent games after injury; however, we noted several players who had a delay in their return-to-play time following their concussions (Figure 3B).

**Figure 4:**
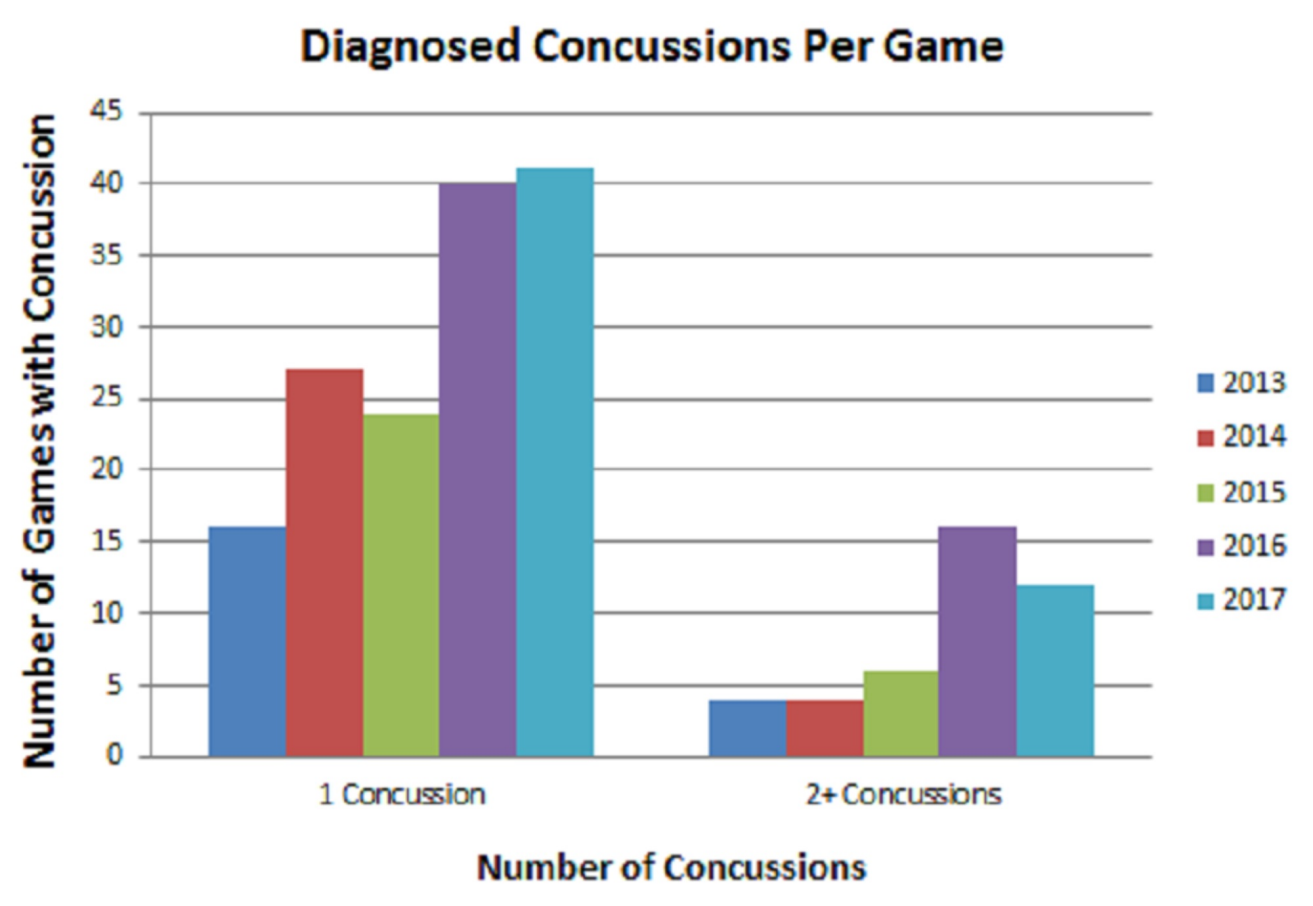
Diagnosed Concussions per Game Bar graphs demonstrating total number of diagnosed concussions per game in which a player was removed to undergo neurological testing

In order to further clarify each seasons concussion rate, we wanted to determine the spread of games over which concussions were diagnosed. Our goal was to observe whether the majority of concussions occurred in a few games or if they were spread across multiple games throughout the full season. We noted the majority of diagnosed concussions occurred on a one per game basis between the 2013 and 2017 seasons. However, our data also showed an increasing trend in the number of games in which multiple concussions were diagnosed between 2013 and 2017 (Figure 4).

**Figure 5:**
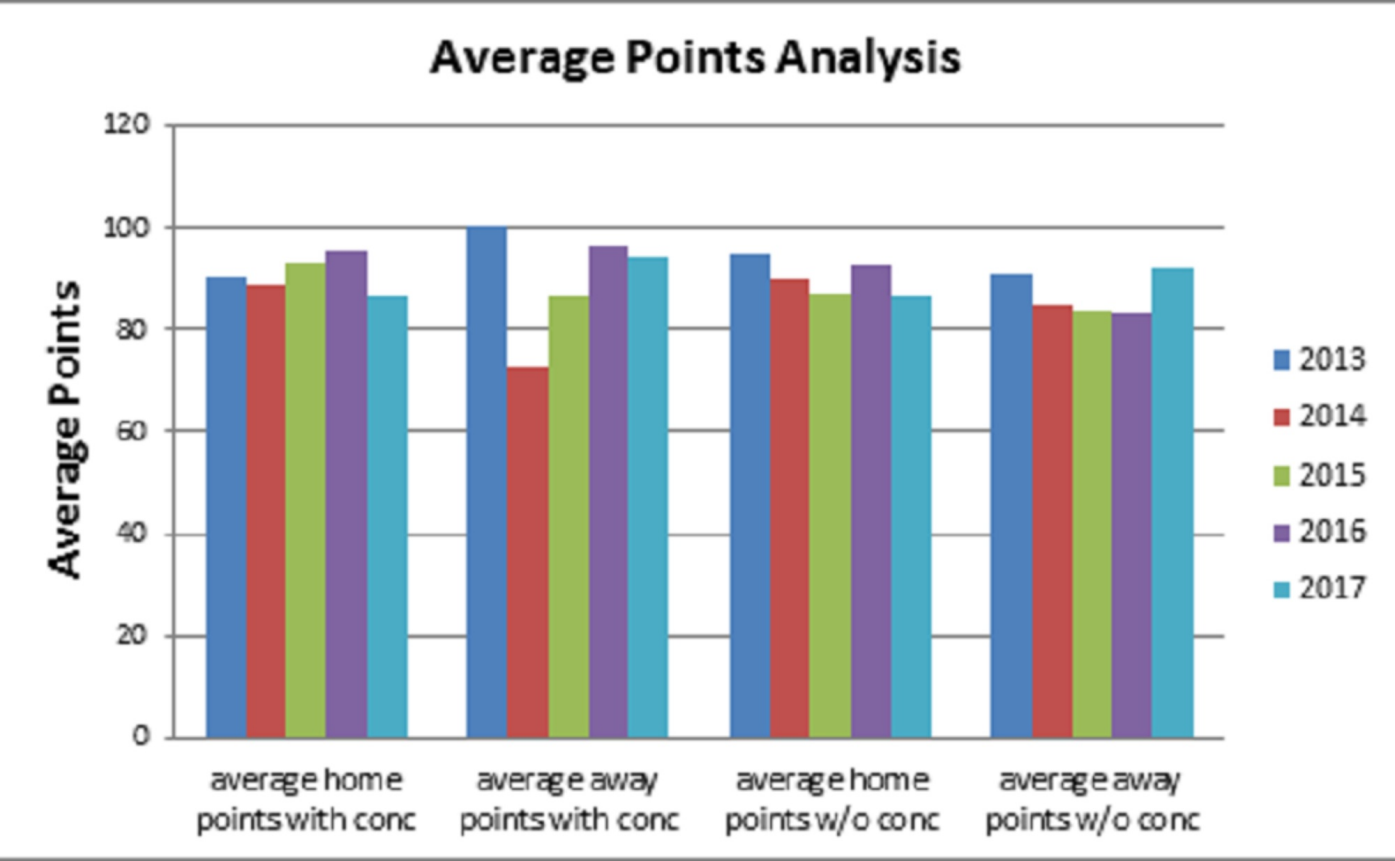
Average Points Scored Analysis Bar graph shows point analysis from the home and away team perspectives, including games with and without concussions for the 2013 (p = 0.5549), 2014 (p = 0.0518), 2015 (p = 0.4239), 2016 (p < 0.0001), 2017 (p = 0.1036) Australian Football League (AFL) seasons. No significance was noted when all seasons were averaged together and compared for games with to without a concussion.

The first style of play analysis performed evaluated the average points scored for both the home and away teams between the 2013 and 2017 AFL seasons. There was no significant difference for the 2013 (p = 0.5549), 2014 (p = 0.0518), 2015 (p = 0.4239), and 2017 (p = 0.1036) AFL seasons when comparing the average points scored in games with or without a concussion. Overall, the trends were found to be non-significant when averaged each individual season together (Figure 5).

**Figure 6:**
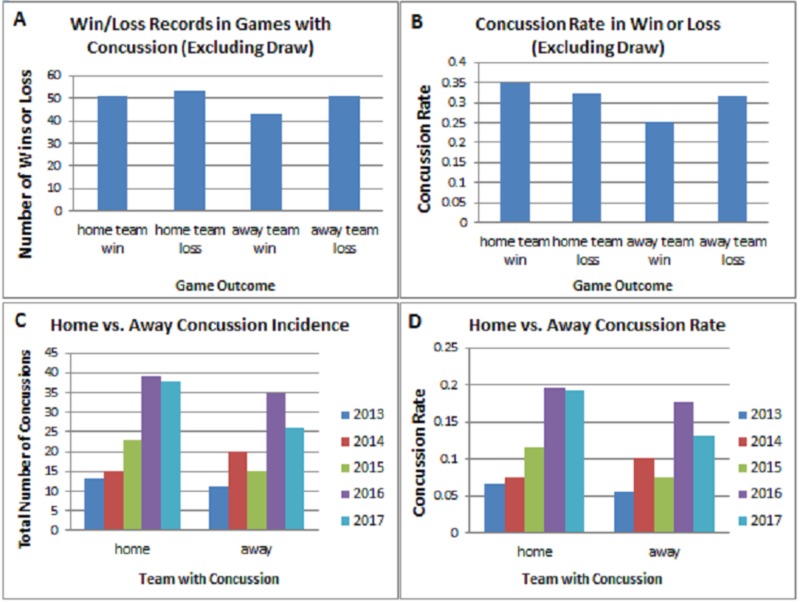
Win/Loss and Home/Away Analysis (A) Bar graph shows average concussion incidence per game when home or away team lost or won games during the 2013-2017 Australian Football League (AFL) seasons. (B) Bar graph showing the concussion rate when home or away team lost or won games during the 2013-2017 AFL seasons. No significant difference was noted in concussion rate for teams that won or lost. (C) Bar graph shows number of concussions sustained by home and away teams during the 2013-2017 AFL season. (D) Evaluation of the concussion rate between the home and away teams. No significance was noted (p = 0.2173).

There was also no significant difference in concussion rate between teams playing from the home or away perspective (p = 0.2173) (Figures 6A-6B). Subsequent to this analysis, we analyzed the implications of a win or loss on concussion rate for the home and away teams across the 2013-2017 seasons. We documented three concussions that occurred when the outcome of the match was a draw, we omitted these from the win/loss analysis in order to not confound the statistics. There was no significant difference in home win versus home loss (p = 0.1182), away win versus away loss (p = 0.2518), home loss versus away win (p = 0.4037), or home win versus away loss (p = 0.2170) (Figures 6C-6D).

**Figure 7:**
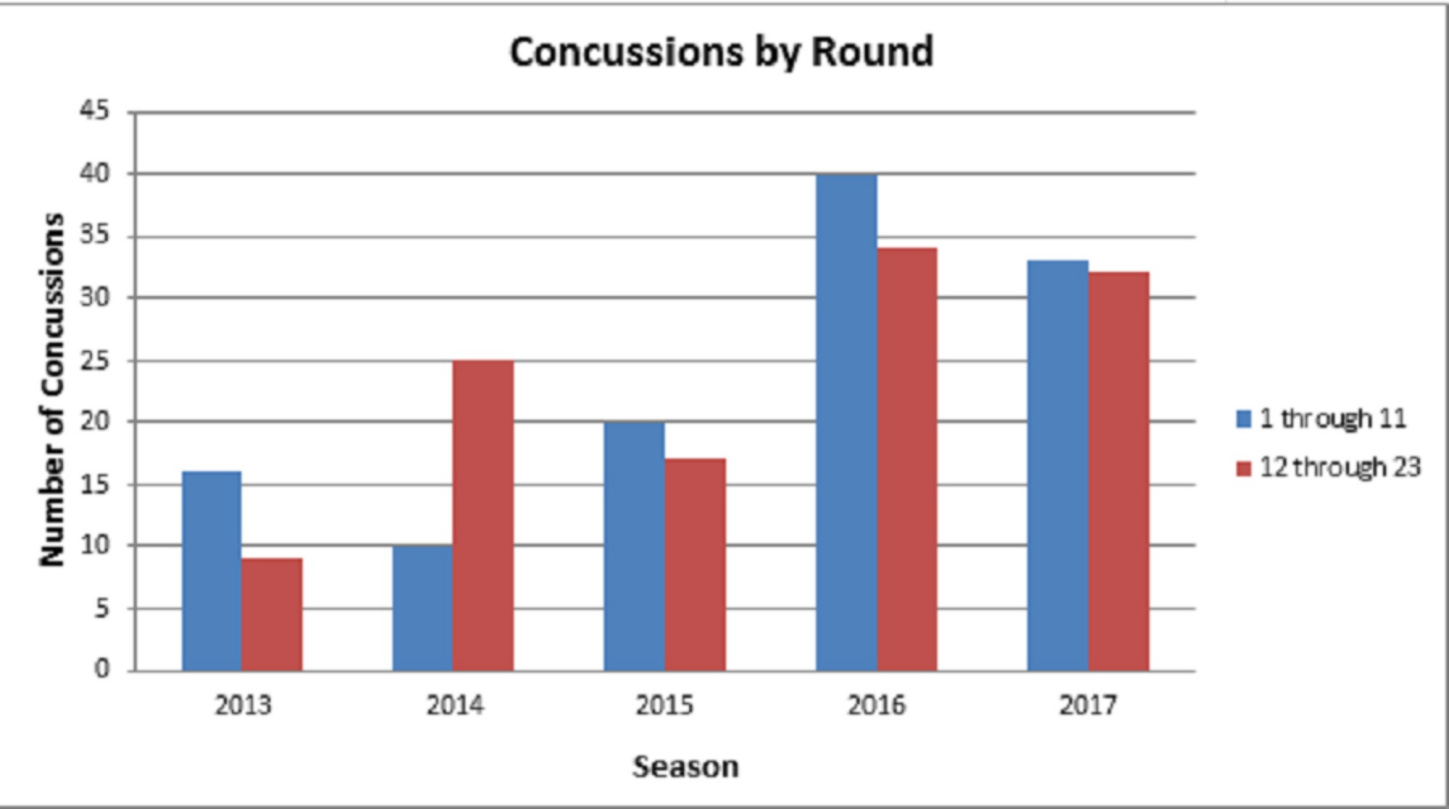
Time of Season Analysis Bar graph shows the number of concussions sustained per season during the first half and second half of the season. No significant difference was noted when comparing Rounds 1-11 to Rounds 12-23 of the combined 2013-2017 season data.

The final analysis evaluated concussion incidence during the time of the season. The 23 rounds of the AFL season were halved in order to assess whether there is any trend in concussion reporting towards the beginning or end of a season. There was no significant difference in the number of concussions reported between the first half (Rounds 1-11) and the second half (Rounds 12-23) for the 2013 (p = 0.2216), 2014 (p = 0.1371), 2015 (p = 0.5487), 2016 (p = 0.4012), and 2017 (p = 0.7004) AFL seasons (Figure 7).

## Discussion

Due to popularization by the media and advances in medical understanding, the long-term risks on head injury and concussion have become more apparent in contact sports. Our study longitudinally examined all 18 professional AFL clubs that competed in the 2013-2017 seasons to gauge the incidence, recurrence, and severity of the concussion. It is the first study to objectively report concussion information from all professional AFLs between the 2013 and 2017 seasons. Our results demonstrated a yearly increase in the cumulative concussion rate of all 18 professional AFL clubs, with a significant difference noted between the 2013 and 2017 seasons (p = 0.0010). It is worth noting that the majority of concussions documented in our dataset occurred on a one per game basis, although we did observe an increasing trend for AFL games in which two or more concussions were diagnosed. This finding can be due in part to multiple modalities surrounding concussion identification and management for a return to play clearance. Prior to the 2015 season, the return-to-play protocol for concussed players was altered. The injured player was required to be removed from the field for 20 minutes of real time instead of 20 minutes of game time in order to allow for adequate time for a team doctor to assess a concussion in the player [10].

To further gauge the magnitude of the concussion, we utilized games missed as a proxy for concussion severity. In previous studies, neuropsychological testing has been used to judge severity. Previous neuropsychological testing of high school and college athletes has shown that deficits in cognitive performance may persist past 14 days (Original Article: Dai J, Li A, Haider S, Tomaselli R, Gometz A, Sobotka S, Post A, Adams R, Maniya A, Lau G, Kaye-Kauderer K, Lovell M, Choudhri T. Modifying Factors for Concussion Incidence and Severity in Professional Football. 2018). Another approach has been to judge concussion severity by the number of games missed after a concussion; more games missed could indicate longer recovery times due to higher concussion severity. Despite this connection, the number of games missed was not as strongly correlated to various factors studied when compared to concussion incidence. We demonstrated a significant increase in average games missed between the 2014 and 2015 AFL seasons (p = 0.0002); however, this increasing trend was not significant when comparing the 2013 to 2017 seasons (p = 0.0951). Our study showed that concussion incidence did not increase as the season progresses, as seen in other high-contact sports studied by our group (Original Article: Dai J, 2018).

While we were unable to acquire source documents directly from the team medical doctors detailing concussion severity, our research team adopted games missed as a proxy for measuring concussion severity. We noted several players who were forced to miss multiple weeks due to a sustained head injury. In 2017, Gibbs et al. performed a 14-year (2000-2013) analysis of a single professional AFL club and found that no players missed the following week’s game due to their concussion [7]. Our results ran contrary to this finding, as we noted several players missing one to two, or more, weeks, after sustaining a head injury. This result may be a product of rule changes to the game and concussion management, which occurred between the two studied time frames. 

Subsequent to the concussion rate and games missed analysis, we evaluated several “style of play” game characteristics to ascertain their effects on concussion incidence. We defined these style of play factors as points scored, win/loss, home/away, and time of season. The results of this analysis indicated no significance across all our “style of play” factors. One noted finding was a significance of <0.0001 in the 2016 points scored analysis for average points in a game with or without concussion. This was deemed to be a product of just that single season's data. When a cumulative average across 2013-2017 seasons was performed, nothing significant was noted for home or away regardless of concussion status.

Limitations

While we consider our data source to be reliable in the reporting of concussions from the AFL, the ideal data set would be comprised of medical diagnoses from the team doctors. It may be possible that our data set is underreporting the total amount of concussions between the 2013 and 2017 AFL seasons. Return-to-play times were not ascertained directly from the team doctor for the clearance date. It may be possible that this data collection modality led to missed cases of head injury or return to play times which could impact the realibility of our dataset.

## Conclusions

Our study identified a significant increasing trend in concussion rate and average games missed that correlates to the data analysis in other high-impact sports such as the NFL and NHL. However, further research is necessary to determine if these findings indicate an improvement in concussion management and player safety measures that is beginning to develop in high-impact sports. We also noted that certain “style of play” factors (points scored, win/loss, home/away, time of season) have no significant implication on concussion rate during the 2013-2017 AFL seasons.

## Figures and Tables

**Figure 8:**
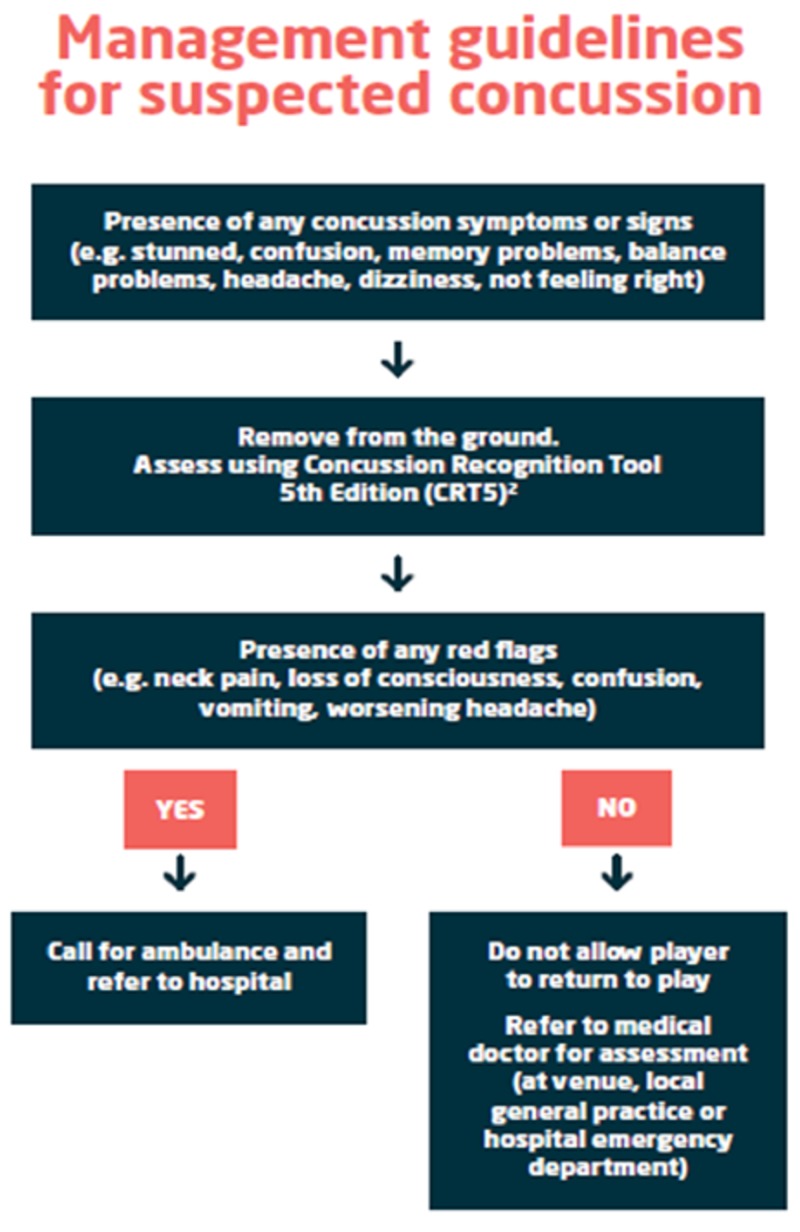
Management Guidelines for Suspected Concussion During the 2017 AFL Season Summary of the management of concussion in Australian Football established in June 2017. http://www.afl.com.au/afleducation/concussion AFL: Australian Football League
